# Supplementary material for: Enhanced Low-Velocity Impact Resistance of Helicoidal Composites by Fused Filament Fabrication (FFF)
Source: Polymers (Basel). 2022 Apr 1;14(7):1440. doi: 10.3390/polym14071440 (PMC9002548; doi:10.3390/polym14071440)
Supplement: Supplementary file 1 [file polymers-14-01440-s001.zip › theoretical prediction.pdf]

The plate is composed of a helical composite material, each layer is isotropic, and each layer rotates at the same angle(α) as the previous one in the vertical direction.

the constitutive equation of the first layer :

$$\{\sigma\} = [D]\{\varepsilon\}.$$

the constitutive equation of the layer of rotation angle :

$$\{\sigma'\} = [T][D][T]^{\mathrm{T}}\{\varepsilon'\}.$$

where $\{\sigma\}$ is the six stress components, σ_x, σ_y, σ_z, τ_{xy}, τ_{yz}, τ_{zx}; $\{\varepsilon\}$ is the six strain components, ε_x, ε_y, ε_z, γ_{xy}, γ_{yz}, γ_{zx}; $[D]$ is the elasticity matrix; and $[T]$ is the angle transformation matrix.

$\{\sigma\}=\{\sigma_x, \sigma_y, \sigma_z, \tau_{xy}, \tau_{yz}, \tau_{zx}\}^T$,

$\{\ \varepsilon\ \}=\{\varepsilon_x, \varepsilon_y, \varepsilon_z, \gamma_{xy}, \gamma_{yz}, \gamma_{zx}\}^T$,

$$[D]=\begin{bmatrix} 1/E & -v/E & -v/E & 0 & 0 & 0 \\ -v/E & 1/E & -v/E & 0 & 0 & 0 \\ -v/E & -v/E & 1/E & 0 & 0 & 0 \\ 0 & 0 & 0 & 1/G & 0 & 0 \\ 0 & 0 & 0 & 0 & 1/G & 1/G \\ 0 & 0 & 0 & 0 & 0 & 0 \end{bmatrix}$$

$$[T]=\begin{bmatrix} \cos\alpha & \sin\alpha & 0 & 0 & 0 & 0 \\ -\sin\alpha & \cos\alpha & 0 & 0 & 0 & 0 \\ 0 & 0 & 1 & 0 & 0 & 0 \\ 0 & 0 & 0 & \cos\alpha & \sin\alpha & 0 \\ 0 & 0 & 0 & -\sin\alpha & \cos\alpha & 0 \\ 0 & 0 & 0 & 0 & 0 & 1 \end{bmatrix}$$

If [D’]=$[T][D][T]$T, $\{\sigma'\}$ = [D’]$\{\varepsilon'\}$.

[D’]=[d_1, d_2,d_3,d_4,d_5,d_6]

$$\{d_1\}=\begin{bmatrix} [\cos\alpha/E+\sin\alpha*(-\nu/E)]*\cos\alpha+[\cos\alpha*(-\nu/E)+\sin\alpha/E]*\sin\alpha \\ [-\sin\alpha/E+\cos\alpha*(-\nu/E)]*\cos\alpha+[-\sin\alpha*(-\nu/E)+\cos\alpha/E]*\sin\alpha \\ (-\nu/E)*\cos\alpha+(-\nu/E)*\sin\alpha \\ 0 \\ 0 \\ 0 \end{bmatrix},$$

$$\{d_2\}=\begin{bmatrix} [\cos\alpha/E+\sin\alpha*(-\nu/E)]*(-\sin\alpha)+[\cos\alpha*(-\nu/E)+\sin\alpha/E]*\cos\alpha \\ [-\sin\alpha/E+\cos\alpha*(-\nu/E)]*(-\sin\alpha)+[-\sin\alpha*(-\nu/E)+\cos\alpha/E]*\cos\alpha \\ (-\nu/E)*(-\sin\alpha)+(-\nu/E)*\cos\alpha \\ 0 \\ 0 \\ 0 \end{bmatrix},$$

$$\{d_3\}=\begin{bmatrix} (-\nu/E)*\cos\alpha+(-\nu/E)*\sin\alpha \\ (-\nu/E)*(-\sin\alpha)+(-\nu/E)*\cos\alpha \\ 1/E \\ 0 \\ 0 \\ 0 \end{bmatrix},$$

$$\{d_4\}=\begin{bmatrix} 0 \\ 0 \\ 0 \\ [\cos\alpha*1/G]*\cos\alpha+[\sin\alpha*1/G]*\sin\alpha \\ [-\sin\alpha*1/G]*\cos\alpha+[\cos\alpha*1/G]*\sin\alpha \\ 0 \end{bmatrix},$$

$$\{d_5\}=\begin{bmatrix} 0 \\ 0 \\ 0 \\ [\cos\alpha*1/G]*(-\sin\alpha)+[\sin\alpha*1/G]*\cos\alpha \\ [-\sin\alpha*1/G]*(-\sin\alpha)+[\cos\alpha*1/G]*\cos\alpha \\ 0 \end{bmatrix},$$

$$\{d_6\}=\begin{bmatrix} 0 \\ 0 \\ 0 \\ 0 \\ 0 \\ 1/G \end{bmatrix}.$$

where E is Young’s Modulus, G is the Shear modulus, v is Poisson's ratio, α is Deflection Angle.
